# Detection of edema in porcine carotid arteries using t2-weighted cardiovascular magnetic resonance

**DOI:** 10.1186/1532-429X-13-S1-P367

**Published:** 2011-02-02

**Authors:** Steen Fjord Pedersen, Won Yong Kim, Samuel Thrysoe, Erling Falk, Steffen Ringgaard, William P Paaske

**Affiliations:** 1 Aarhus University Hospital, Aarhus, Denmark

## Introduction

Arterial wall inflammation destabilizes atherosclerotic plaques and makes them prone to rupture (1) so detection of inflammatory activity may therefore assist discrimination between unstable and stable plaques. Inflammation of the vessel wall prompts transport of excess fluid into the interstitial space and results in the formation of edema (2). Hence, edema located within the vessel wall can be used as a marker for inflammatory activity.

## Purpose

We wished to determine whether edema could be detected in the carotid artery wall by cardiovascular magnetic resonance (CMR) using a T2-weighted short-tau inversion recovery sequence (T2-STIR).

## Methods

An overstretched balloon injury was induced unilaterally in the common carotid arteries of six pigs to create inflammatory edema in the artery wall. Post injury, T2-STIR (TR/TE/TI: 1600/100/100 msec) was performed on two cross sections of the injured as well as the contralateral, uninjured carotid artery using a 1.5 T MRI scanner. CMR images were matched to the corresponding histopathology. They were checked against the occurrence of Evans blue and correlated to the amount of fibrinogen in the vessel wall that was used as a surrogate marker for edema.

## Results

**Figure 1:**
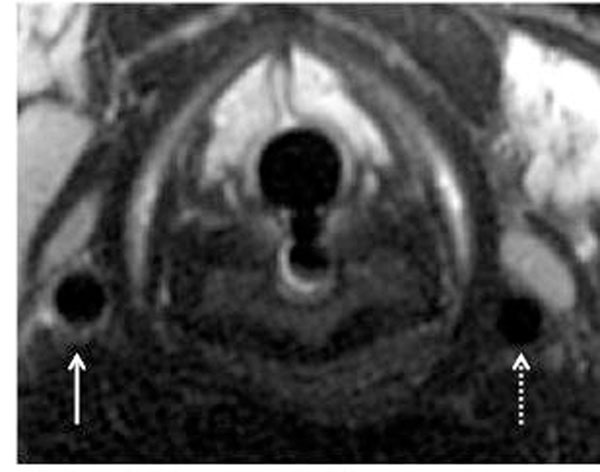
T2-STIR cross-sectional image of the carotid arteries from a single MRI examination four days post injury. Note that there is a large difference in SI between the injured (non-stippled arrow) and uninjured artery (stippled arrow).

**Figure 2:**
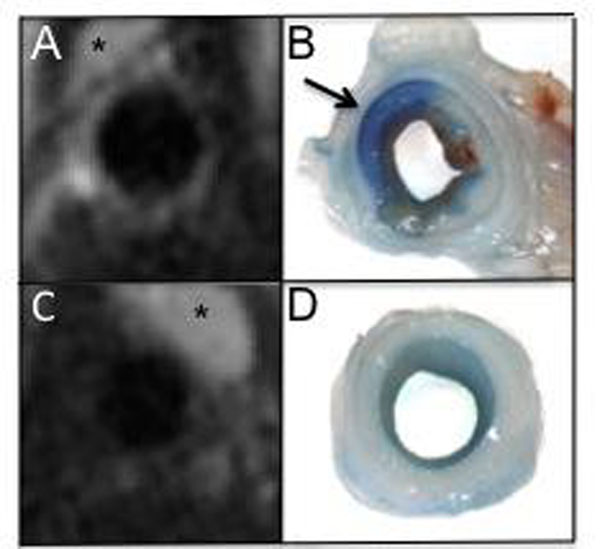
T2-STIR weighted CMR images of balloon injured (A) and uninjured (C) carotid artery, notice the strong SI of the injured artery wall. Photos of the corresponding segments demonstrate that there is uptake of Evans blue in the injured arterial wall (arrow) (B) and no uptake in the uninjured wall (D). *indicates thymus that has a high SI on T2-STIR.

**Figure 3:**
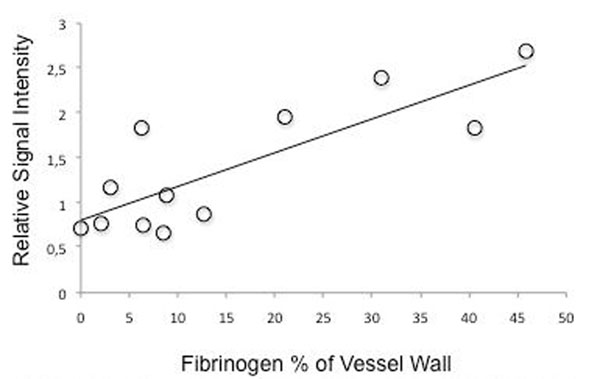
Mean fibrinogen staining of the injured and uninjured carotid artery segments versus mean relative SI on the corresponding T2-STIR images (r=0.80; p<0.001).

**Figure 4:**
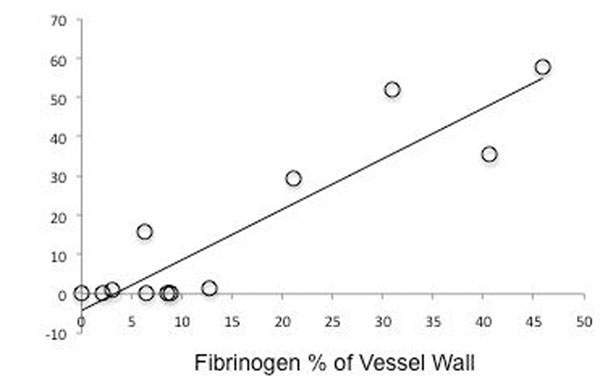
Mean fibrinogen staining of the injured and uninjured carotid artery segments versus main area of enhancement on the corresponding T2-STIR images (r=0.93; p<0.001).

The relative signal intensity (SI) of the injured carotid artery was 233% (p<0.001; CI95 = [185%-282%]) higher than the uninjured control carotid artery (Fig. 1). Agreement was detected between carotid artery wall enhancement (defined as SI four SD ≥ the sternocleidomastoid muscle) and the uptake of Evans blue (κ=0.83, p<0.001) (Fig. 2). The relative signal intensity and area of enhancement showed a linear correlation with the area of fibrinogen detected on the corresponding histopathology (r=0.80, p<0.001) and (r=0.90, p<0.001), respectively (Figs. 3 and 4). The T2-STIR sequence detected edema in the vessel wall (i.e., enhancement) with a sensitivity of 100 and a specificity of 75.

## Conclusion

T2-STIR enabled detection of edema in the carotid artery wall and clear discrimination between uninjured and injured carotid artery. These findings imply that T2- STIR has the potential to become a non-invasive diagnostic tool for the detection of inflammatory activity in carotid arteries.
